# Clinical characteristics and associating risk factors of gastrointestinal perforation in children with IgA vasculitis

**DOI:** 10.1080/07853890.2021.2009554

**Published:** 2021-12-08

**Authors:** Qingyin Guo, Xiaolei Hu, Chundong Song, Xianqing Ren, Wensheng Zhai, Ying Ding, Xia Zhang, Meng Yang, Jian Zhang, Miao Jiang

**Affiliations:** a First Affiliated Hospital of Henan University of Chinese Medicine, Henan Children's Hospital of Integrated Traditional Chinese and Western Medicine, Zhengzhou, China; b Henan University of Chinese Medicine, Zhengzhou, China

**Keywords:** Children, IgA vasculitis, gastrointestinal perforation, retrospective analysis, risk factors

## Abstract

**Background:**

IgA vasculitis (IgAV) is a common small vessel vasculitis in children. Gastrointestinal perforation (GP) rarely presents as a complication of IgAV and is not well characterized. This study is aimed to investigate the clinical features, diagnosis, and risk factors of GP in children with IgAV.

**Methods:**

We retrospectively reviewed the clinical data of children with IgAV who attended our hospital between January 2014 and June 2018. The clinical risk factors and the corresponding treatments were analyzed for the children with IgAV complication with GP.

**Results:**

In total, 10,791 children with IgAV were reviewed in this study. GP was observed in 11 children with IgAV, accounted for 0.10% of the total cases. Among those GP patients, 1 case was gastric perforation, 10 cases were intestinal perforation. Five GP cases were identified by abdominal CT. Ultrasonography was failed to detect the occurrence of GP in five cases. The average duration of abdominal pain in the GP cases was 9.3 days, and 9 cases (81.8%) presented with abdominal pain for over 7 days. Gastric/intestinal perforation repair were performed for 3 IgAV GP cases under open surgery. The other eight cases were treated through enterectomy. In comparison with the patients without GP, the GP patients had significant higher rates in the aspect of the abdominal or mixed type of IgAV, abdominal pain duration more than 7 days, hematochezia, renal damage, and methylprednisolone treatment with the daily dosage more than 2 mg/kg.

**Conclusion:**

GP children accounted for 0.10% of the total IgAV cases. The risk of GP is elevated in IgAV patients who has gastrointestinal symptoms and/or other symptoms such as hematochezia, renal damage, a prolonged abdominal pain (>7 days), administration of methylprednisolone (>2 mg/kg). Abdominal CT is highly recommended for the early detection of GP in IgAV patients.

**Key messages**
- Gastrointestinal perforation (GP) rarely presents as a complication of IgAV and is not well characterized.
- 11 out of 10,791 children with IgAV developed GP, accounting for 0.10% of the total number of cases.
- Abdominal CT is highly recommended for the early detection of GP in IgAV patients.

## Introduction

IgA vasculitis (IgAV) is a systemic vasculitis based on extensive inflammation of small blood vessels and is the most common vasculitis in childhood [1]. The incidence of IgAV was 20.4 per 100,000 [2]. IgAV patients may have some complications, including blistering eruptions [3], pancreato-biliary involvement [4], hepatitis [5], and gastrointestinal involvement [6]. Clinical manifestations of IgAV include palpable skin purpura, gastrointestinal symptoms, joint symptoms, and renal damage. IgAV patients mainly represented with gastrointestinal symptoms is called abdominal type IgAV. In severe cases, intestinal obstruction and intestinal perforation may occur and could be life-threatening if there is no immediately medical intervention [7,8]. The clinical characters of IgAV complicated with GP has not been well studied, although it has been reported through a few case studies [9–11]. Studies with larger clinical data are needed to establish the relationship between the risk factor and the occurrence of GP in IgAV patients. We here retrospectively analyzed 11 cases of IgAV with GP among 10,791 IgAV patients in terms of clinical characteristics, diagnosis, treatment and risk factors.

## Methods

A total of 10,791 cases (aged 1–17 years) of children with IgAV who attended the Department of Paediatrics from January 2014 to June 2018 were selected as study subjects. The diagnosis of IgAV was based on the European league of rheumatology (EULAR) and the children's rheumatology international (PRINTO) and the children's rheumatology league (PRES) criteria in 2010 [12]. The clinical data of 11 children with GP (perforation group) were collected, including gender, age, clinical manifestations, abdominal pain duration, hematochezia, renal damage (proteinuria or haematuria (proteinuria >0.3 g/24 h, haematuria or red blood cell casts: >5 red blood cells/high power field), diagnosis, and treatment. Forty-two of the 10,780 IgAV children without GP were randomly selected (whose hospital admission number includes the number “111,” such as xxx111xx, xxxxx111, etc.) as the control group. The risk factors that may affect GP in children with IgAV were analyzed including gender, age, IgAV type, abdominal pain duration, hematochezia, and renal damage. All the clinical data used in this study were obtained from hard copies and electronic medical records and approved by the medical ethics committee of the First Affiliated Hospital of Henan University of traditional Chinese medicine.

## IgAV clinical classification

The classification of IgAV was divided into:

Skin type: presented with simple skin purpura only; joint type: in addition to skin purpura, joint swelling, and pain symptoms; abdominal type: presented with gastrointestinal symptoms and signs, such as abdominal pain nausea, and vomiting in addition to skin purpura; renal type: skin purpura accompanied by haematuria and/or proteinuria specific; mixed type: in addition to skin purpura, the other three types of two or more.

## Statistical analysis

The descriptive analysis included calculations of various proportions. To explore the risk factors of GP in IgAV children, 11 cases IgAV children with GP as a perforated group, 42 cases from the remained 10,780 cases IgAV children were selected as control unperforated group. Chi-squared test and univariate logistic regression were used to analyze the risk factors that might affect the occurrence of GP in IgAV children, such as gender, age, purpura type, the time of abdominal pain, hematochezia, renal damage, methylprednisolone dose more than 2 mg/kg. Statistical analyses were performed using SPSS statistical package 22.0 (IBM SPSS Inc., Chicago, IL). A value of *p* < .05 was considered statistically significant.

## Results

### Clinical manifestations of children with IgAV

**Figure 1.**
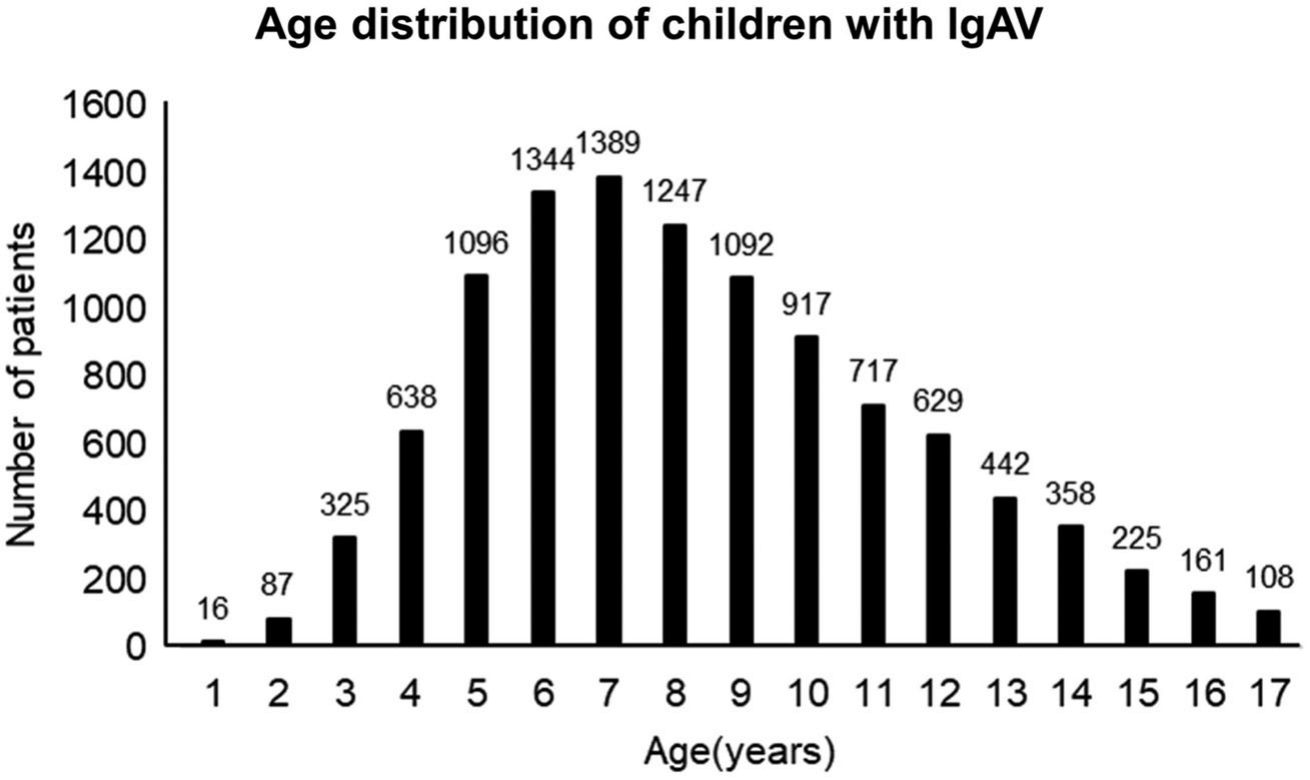
Age distribution of children with IgAV.

A total of 10,791 cases of children with IgAV attended the department of paediatrics between January 2014 and June 2018, including 6278 males and 4513 females. The average age of onset was 8.33 ± 3.23 years. IgAV mainly occurred in children aged between 3 and 14 years, with a total of 10,194 (94.47%) cases. The peak morbidity age was 5–9 years, with a total of 6168 (57.16%) cases. The results are shown in Figure 1. Based on the clinical manifestations and laboratory tests, the IgAV patients were divided into the skin, the joint, the abdominal, the renal, and the mixed type (Table 1). Among 10791 IgAV children, 4357 cases (40.75%) with abdominal symptoms.

**Table 1.** Clinical manifestations of children with lgAV.

Clinical manifestation	All	Skin	Joint	Abdominal	Renal	Mixed			
						Abdominal + Renal	Abdominal + Joint	Joint + Renal	Abdominal + Renal + Joint
No. of cases	10,791	1992	2206	1523	1715	1117	988	511	729
Age, y	8.33 (1–17)	8.92 (1–17)	7.56 (1–17)	8.06 (1–17)	9.71 (2–17)	7.91 (1–17)			
Sex, ♂:♀	6278:4513	1090:902	1249:957	895:628	1071:644	1972:1382			
Percent（%）	100	18.46	20.44	14.11	15.89	10.35	9.16	4.73	6.75

### Clinical characteristics and diagnosis of GP in children with IgAV

**Figure 2.**
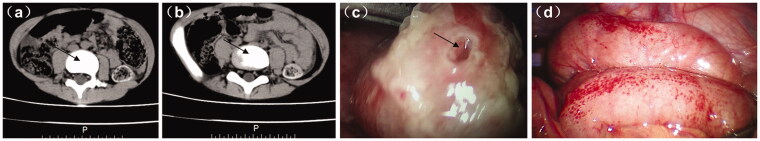
CT diagnosis and treatment of IgAV complicated with GP. (a and b) CT image of IgAV complicated with GP (case 6); (c) intraoperative image of IgAV complicated with GP (case 6). Arrows indicate perforation of the digestive tract and (d) multiple bleeding points in intestinal mucosa (case 11).

A total of 11 out of the 10,791 IgAV children developed GP. The 11 GP children, 8 males and 3 females, aged 5–13 years, with an average age of 8.09 years. These 11 GP children were accompanied with massive purpura, haematemesis, and gastrointestinal symptoms, including facial skin purpura found in 3 cases, joint pain presented in 8 cases, and renal damage developed in 7 cases. The perforation was found in the gastric (1 case) and intestinal (10 cases) tract. Ileocecal region, terminal ileum and ileum counted for 4 cases, 1 case and 5 cases, respectively. At the time of diagnosis of GP in these patients, abdominal pain had lasted from 3 to 14 days, an average of 9.3 days. 3 GP cases were diagnosed by colour doppler ultrasound without CT, 8 GP cases were diagnosed by CT. Case 1 (gastric perforation), case 6 (terminal ileum perforation), and case 11 (ileum perforation), had a small perforation and received gastric/intestinal repair due to early detection, and the remaining 8 patients underwent enterectomy. Case 7 had a small intestinal diverticulum and underwent diverticular resection due to severe abdominal pollution. Cases 4 and 5 underwent intestinal resection and anastomosis plus intraperitoneal fistula. And the fistula was pulled out half a year later. Among the 11 cases, there was no death and no postoperative complications such as intussusception and intestinal adhesion. Renal damage occurred in 7 cases, all of them were treated with drugs and had no serious gastrointestinal symptoms. 4 cases were cured without skin purpura and gastrointestinal symptoms. The diagnosis and treatment of the children with GP are shown in Table 2. CT diagnosis and surgical treatment of GP in children with IgAV are shown in Figure 2.

**Table 2.** Diagnosis and treatment of lgAV complicated with GP.

Diagnosis	Treatment method	No. of cases	Age (years)	Days of abdominal pain	Methylprednisolone dose
Gastric perforation	Intestinal perforation repair	1	7–13	16	3。 mg/kg/day
Intestinal perforation	Intestinal perforation repair	2	7–13	9–11	2.8–3.0 mg/kg/day
	Enterectomy	5	1–13	3–16	2.4–3.2 mg/kg/day
	Enterectomy + fistula	2	1–13	7–11	2.5-3.0 mg/kg/day
	Enterectomy + diverticulectomy	1	7–13	11	2.3 mg/kg/day

### Risk factors analysis of GP in children with IgAV

We then explored the potential risk factors of GP in IgAV children. As the results in Table 3 showed, there was no significant difference in age and gender between the two groups. The abdominal type (or mixed type) IgAV, hematochezia, renal damage, abdominal pain duration more than 7 days, methylprednisolone dose more than 2 mg/kg showed statistically significant differences (*p <* .05) (Table 3) between the two groups. As only 11 cases of GP occurred in this study, multivariate regression analysis cannot be performed, the reliability of these results still needs to be confirmed by further studies.

**Table 3.** The univariate *Chi*-*square* test of risk factors of lgAV with GP [*n* (%)].

Group	Case number	Age ≤6 years	Male	Abdominal (mixed) IgAV	Hematochezia	Renal damage	Abdominal pain duration more than 7 days	Methylprednisolone dose more than 2 mg/kg
Perforated group	11	3 (27.3)	8 (72.7)	11 (100.0)	11 (100.0)	7 (63.6)	9 (81.8)	11 (100)
Control	42	19 (54.3)	28 (66.7)	27 (64.3)	3 (7.14)	11 (26.2)	0 (0)	3 (7.14)
*χ*^2^ value		1.159	0.147	5.479	38.668	5.449	41.39	38.67
*p* value		.282	.701	.019	5.02284E-10	.019	1.2453E-10	5.02284E-10

## Discussion

IgAV is a common vasculitis in childhood [1]. Gastrointestinal symptoms were frequently observed in children with IgAV, accounting for 50–75% [2,7,13], abdominal distention and abdominal pain are the usual clinical manifestations, and the pathological manifestations are erosion, edoema, and necrosis of gastrointestinal tract mucosa [14]. Typical endoscopic manifestations of IgAV include diffuse mucosal edoema, erythema, and ecchymosis, or multiple irregular ulcers [15,16]. In our study, 11 of the 10,791 IgAV children developed GP, accounted for 0.10% of the total IgAV cases, lower than the previous report of 0.38% [17]. To our knowledge, this study represents the biggest retrospective case series hitherto published.

GP is one of the abdominal complications requiring surgical treatment in IgAV children. Other complications include intussusception, massive gastrointestinal bleeding and intestinal necrosis [18]. The symptoms of children with GP are relatively serious, and delayed treatment may endanger the life of the patients. Hence, we analyzed the possible risk factors of GP in IgAV children, including age, gender, purpura type, duration of abdominal pain, hematochezia, renal damage, methylprednisolone dose more than 2 mg/kg.

In this study, 7 IgAV children with GP were associated with renal damage (63.63%), which was significantly higher than that of the control group (26.2%), suggesting that IgAV children with renal damage had a greater risk of GP. Some studies indicate that abdominal pain may be one of the important risk factors of IgAV children's renal damage [9,19], therefore, IgAV children with gastrointestinal symptoms should be regularly monitored with a routine urine test, renal function assessment. All of the 11 GP IgAV showed hematochezia (100%) versus only 3 cases (7.14%) in the control group, which indicates that hematochezia is a risk factor of GP. In addition, GP cases manifested a significant amount of skin purpura including 3 cases of facial purpura. However, whether the severity and position of skin purpura can be used as an indicator for early diagnosis of GP still needs further research.

The pathogenesis of GP in IgAV children is still not fully understood. Causes of GP may include diverticulitis, gastric ulcer, and intestinal ischaemia [20]. It was reported that thrombus caused by vasculitis may cause intestinal ischaemia, followed by necrosis and perforation of the intestinal wall [21]. Ultrasonography or CT signs are useful to confirm the diagnosis of GP. The key ultrasonographic finding of gastrointestinal perforation is the free gas under the diaphragm and a small amount of gas and fluid next to the perforation. When the perforation is small, the subphrenic gas is less, or the intestinal perforation is wrapped by other surrounding tissue so that the gas cannot reach the subdiaphragm. Or the patient has a large amount of air in the intestinal loop, which affects the colour ultrasound diagnosis. In our study, 5 patients did not show perforation under ultrasonography, and was diagnosed by CT subsequently. Therefore, GP should not be ruled out with a negative ultrasonography finding. CT examination is highly recommended for GP diagnosis.

In our study, the average duration of abdominal pain of GP in IgAV children was 9.3 days, which is consistent with the previous report that intestinal perforation of IgAV occurred during the second week after steroids used [17]. There are 9 cases (81.8%) of GP children abdominal pain duration more than 7 days, while the longest time in the control group is 5 days. The duration of abdominal pain in IgAV children more than 7 days can be considered as an early sign of GP occurrence. Glucocorticoid can reduce edoema, relieve pain, and reduce the risk of surgical intervention in an early stage of GP [8, 19]. Unfortunately, systemic glucocorticoid therapy may mask symptoms of surgical complications such as abdominal pain and fever [19]. Some studies have shown that large doses of corticosteroids can reduce the synthesis of mucus in the gastrointestinal mucosa, inhibit the regeneration and healing of the intestinal mucosa, reduce the thickness of the gastrointestinal wall, all of which increase the risk of intestinal perforation [9,10,17,22]. Our study indicated that a course of methylprednisolone of more than 7 days and a dose of more than 2 mg/kg were risk factors of GP in IgAV children. However, hormones are still the most effective treatment options for abdominal symptoms of IgAV. Therefore, in the clinical treatment of IgAV patients, it is necessary to weigh the advantage and disadvantages of the high-dose and long-term application of hormone.

## Conclusions

IgAV children with severe gastrointestinal symptoms should be evaluated for GP. In the current study, we found that the abdominal or mixed type of IgAV, abdominal pain duration more than 7 days, hematochezia, renal damage, methylprednisolone dose more than 2 mg/kg were associated with the risk of GP in IgAV children. It is necessary to cooperate with physical examination and combined with colour ultrasound or CT examination to identify and diagnose IgAV-related gastrointestinal tract abnormalities as early as possible to give timely and appropriate surgical intervention. Due to GP is relatively rare in IgAV children, the number of cases can be expanded or a multicenter clinical study can be conducted to further study the relationship between IgAV and GP.

## Data Availability

The data used in this study are available upon request of the author Qingyin Guo. The paper and electronic medical record used in this study belong to the First Affiliated Hospital of Henan University of Chinese Medicine and is available only *via* administrative permission.

## Abbreviations

CT: computerized tomography
GP: gastrointestinal perforation
IgAV: IgA vasculitis
